# Phylogenetic Diversity Outperforms Functional Diversity in Predicting Aboveground Biomass Across Tree Strata in a Subtropical Forest

**DOI:** 10.1002/ece3.73774

**Published:** 2026-06-04

**Authors:** Qingping Li, Yizhi Wang, Xiuqin Ci, Zhiyun Lu, Yuanjie Xu

**Affiliations:** ^1^ College of Soil and Water Conservation Southwest Forestry University Kunming China; ^2^ Forestry College of Southwest Forestry University Kunming China; ^3^ Centre for Integrative Conservation, Xishuangbanna Tropical Botanical Garden Chinese Academy of Sciences Menglun China; ^4^ Key Laboratory of Tropical Forest Ecology, Xishuangbanna Tropical Botanical Garden Chinese Academy of Sciences Menglun China; ^5^ Ailaoshan Station for Subtropical Forest Ecosystem Studies Chinese Academy of Sciences Jingdong China; ^6^ Zhanyi Karst Ecosystem Observation and Research Station Kunming China

**Keywords:** aboveground biomass, functional diversity, functional identity, phylogenetic diversity, tree layers

## Abstract

Forest aboveground biomass (AGB) is crucial for maintaining global carbon balance, with its accumulation closely associated with biodiversity. However, the mechanisms by which these diversity effects take place, and which dimension of biodiversity that has the most prominent impact on AGB productivity remain insufficiently elucidated. We measured AGB, functional traits and evolutionary relatedness of the overstory and understory tree layers in a subtropical forest in Yunnan Province, China. We assessed predictive importance of functional and phylogenetic diversity indices on AGB across tree layers by using partial least squares (PLS) regression. Overstory exhibited greater AGB with tree species showing higher community‐weighted means of resource‐acquisitive traits (e.g., specific leaf area), as well as higher phylogenetic diversity. The understory AGB was mainly enhanced by conservative strategies (e.g., lower specific leaf area) as well as mean nearest taxon distance; and it was also increased by higher phylogenetic species richness and functional richness. Our results highlight the role of functional identity in regulating AGB. Notably, contrary to the results in previous Biodiversity and Ecosystem Function (BEF) studies where the influence of functional diversity was greater than phylogenetic diversity, we found that phylogenetic diversity is more capable of explaining the accumulation of AGB than functional diversity across tree strata. Our study thus underscores the significance of incorporating phylogenetic diversity into biodiversity and ecosystem function studies.

## Graphical abstract

Conducted in a subtropical forest in southwestern China, this study evaluates how various dimensions of plant biodiversity regulate aboveground biomass (AGB) across forest strata under consistent edaphic and topographic conditions, addressing the insufficiently elucidated question of which biodiversity dimension most prominently impacts AGB productivity. It reveals that tree species with acquisitive strategies in the overstory and those with conservative strategies in the understory respectively promote AGB accumulation, a key finding linking tree functional strategies to AGB dynamics across different forest layers. Importantly, phylogenetic diversity outperforms functional diversity in explaining AGB accumulation across tree strata, which provides evidence opposite to most existing empirical studies and serves as a valuable supplement to research on how biodiversity affects AGB in subtropical forests.

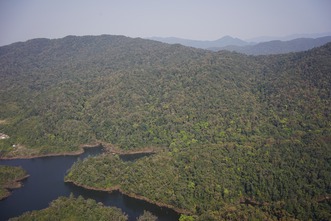

## 1 Introduction

Forest aboveground biomass (AGB) is fundamental for estimating aboveground carbon storage and understanding carbon balance (Behera et al. 2017). Enhancing forest carbon sequestration has emerged as a vital strategy to mitigate the rising atmospheric CO_2_ concentrations and address climate change (Lewis et al. 2019). Carbon sequestration, a key ecosystem function, is largely regulated by biodiversity in addition to being determined by environmental conditions (Isbell et al. 2018). Continued loss of biodiversity can disrupt the balance of resource utilization within communities. This may be reflected in the form of decreasing species numbers or biotic homogenization (Kramer et al. 2023; Dehling and Dehling 2023). While species loss leads to niche vacancies, biotic homogenization may weaken the adaptability of communities to environmental fluctuations (Torres et al. 2024; Hessen 2024). These changes ultimately lead to resource underutilization and reduce biomass production efficiency. Consistent evidence suggests that ecosystem functions such as AGB or productivity generally exhibit positive responses to increased biodiversity under controlled experimental conditions (Hong et al. 2022; Veryard et al. 2023). Therefore, understanding the underlying mechanisms by which biodiversity influences AGB is essential for addressing the challenge of rising atmospheric CO_2_ concentrations (Díaz et al. 2011; Ali 2023).

In natural forests, species‐rich communities promote AGB accumulation through two critical pathways: first, diverse plant traits shape differentiated resource uptake strategies; second, functional complementarity among species improves overall efficiency of resource utilization (Hooper et al. 2005; Li et al. 2018; Lü et al. 2019). It is reported that species‐rich forests often exhibit higher phenological asynchrony (both interspecific and intraspecific), allowing communities to achieve optimal utilization of resource at longer timescales, substantially reducing competitive pressure among species, thereby enhancing AGB (Zuppinger‐Dingley et al. 2014). However, as a commonly used predictor for ecosystem functioning, species diversity mainly reflects the richness and evenness of species (Roswell et al. 2021). The relevant information about interspecific trait variation and niche complementarity in plant communities still needs to be represented by community‐weighted mean (CWM) traits and functional diversity (Vargas‐Larreta et al. 2021). These diversity indices are often linked to two mechanisms explaining diversity effects. When AGB is driven by CWMs, it suggests that specific traits of dominant species regulate biomass accumulation (Cadotte 2017), supporting the selection effect or mass ratio hypothesis. Conversely, if functional diversity drives AGB production, then the complementarity effect is more explanatory. Beyond functional diversity, phylogenetic diversity is increasingly recognized as an indicator of niche complementarity recently (Chen et al. 2025).

In natural communities, species coexistence is jointly shaped by functional traits and evolutionary history (Cavender‐Bares et al. 2009). To date, a number of studies have suggested that functional diversity exhibits greater explanatory power than phylogenetic diversity in predicting variations in ecosystem functioning (Flynn et al. 2011; Steudel et al. 2016). In forests of southwestern China, functional diversity was found to be significantly and negatively associated with forest productivity, whereas phylogenetic diversity showed no significant effect (Zou et al. 2024). Nevertheless, given that phylogenetic diversity may serve as a proxy for cryptic functional traits closely related to ecosystem functioning, it possesses greater explanatory power than other biodiversity metrics (Cadotte et al. 2008; van der Plas 2019; Wang et al. 2026). Phylogenetic diversity measures the total evolutionary distance among coexisting species, reflecting the evolutionary history within a community (Srivastava et al. 2012). Moreover, since phylogenetically close species often share similar functional traits and niches (Ackerly 2009), phylogenetic diversity not only serves as an indicator of niche partitioning but is also regarded as a key driver of complementarity effects (Chen et al. 2025). Empirically, greater phylogenetic distance among species is associated with reduced resource overlap and stronger complementarity (M. W. Cadotte 2013). Recent studies have reported significant associations between phylogenetic diversity and ecosystem processes such as carbon stock and nutrient cycling (Cadotte et al. 2009; Mensah et al. 2024; Tang et al. 2023).

Subtropical forests are characterized by high productivity and complex stand structures compared with other ecosystems (Wu et al. 2025). This structural complexity stems from marked variation in tree size distributions and spatial heterogeneity of canopy architecture (Ray et al. 2023). In the whole community, tree species vary in statures and life‐forms, and those in the same synusia possess comparable life‐forms and environmental associations (Richards 1971). Ultimately, species composition and diameter‐class diversity determine forest strata structure and underpin their capacity for resource acquisition (Ehbrecht et al. 2021). Within such forest structures, the majority of community‐level AGB is concentrated in the overstory, with the understory contributing relatively less to total biomass pool (Oberle et al. 2009; Zhang et al. 2016); thus, the understory remains largely overlooked. Nevertheless, understory biomass is a key indicator of forest ecosystem functions, as understory vegetation influences tree regeneration and growth by competing with overstory trees for essential resources (Landuyt et al. 2020). Furthermore, the understory layer makes significant contributions to the floristic diversity of forests (Halpern and Spies 1995), influences the trajectory and pace of forest succession (Royo and Carson 2006), and exerts a vital impact on energy flow and nutrient cycling (Nilsson and Wardle 2005). As these functions are closely associated with understory biomass (Gilliam 2007), it is essential to consider both the overstory and understory layers to comprehensively understand the process of AGB accumulation in subtropical forests. This study aims to explore the underlying mechanism of diversity effects on AGB from multiple facets (including functional identity, functional diversity and phylogenetic diversity) across tree layers within shared habitats under the same edaphic and topographic conditions. We hypothesize that (1) owing to the dissimilarities in resource acquisition strategies between strata, functional identity may influence AGB in both layers, but the specific functional traits of each layer underpinning mass ratio hypothesis are likely to vary between overstory and understory; and (2) phylogenetic diversity explains AGB more effectively than functional diversity via complementarity effects, as it captures phylogenetically conserved traits that are difficult to measure directly (e.g., defense traits) (Castagneyrol et al. 2014; Hähn et al. 2025).

## 2 Materials and Methods

### 2.1 Study Area

**FIGURE 1:**
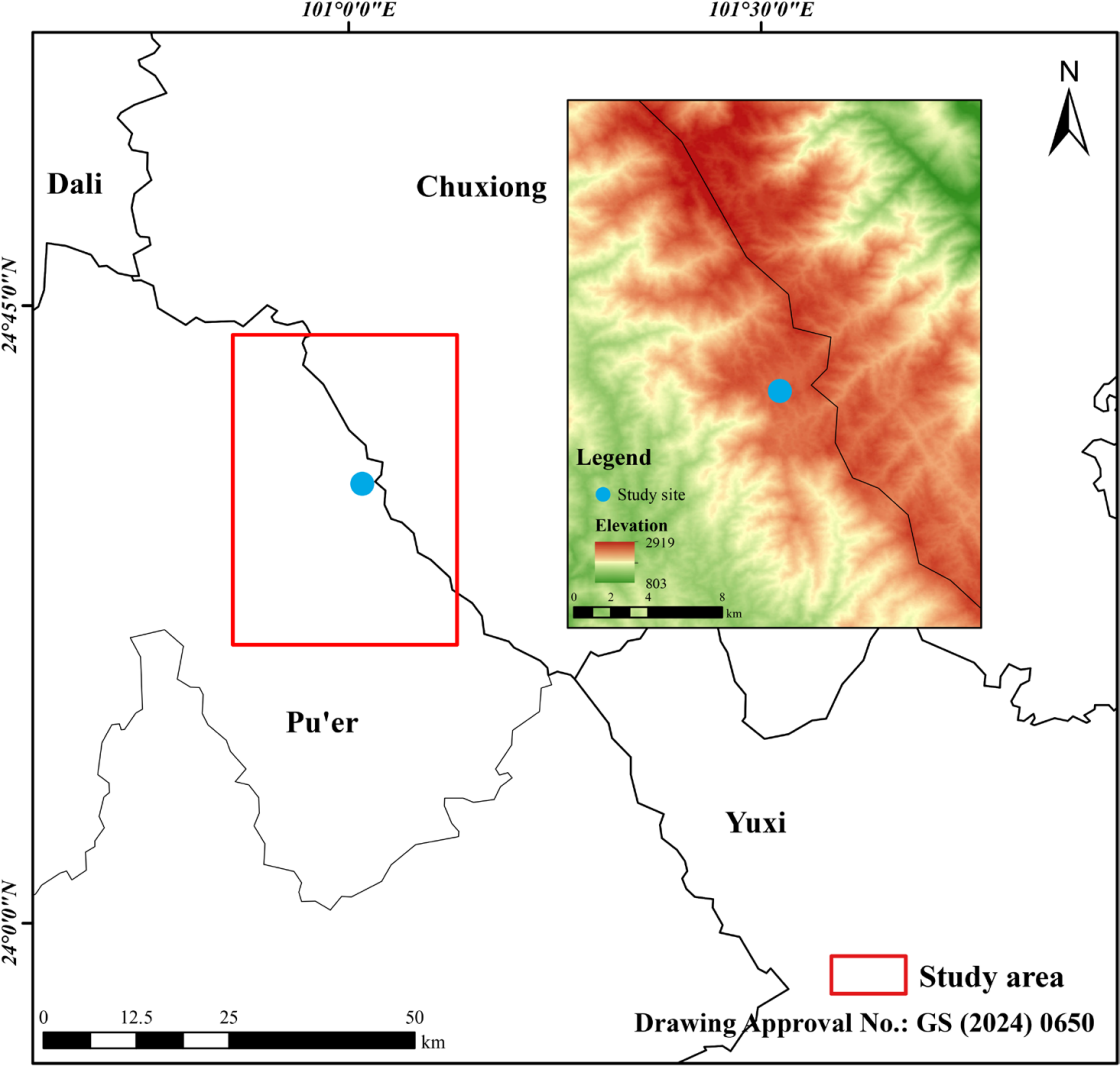
Location of the study area.

This study was conducted in the Ailao Mountains National Nature Reserve (24°32′N, 101°01′E), located in the southern Hengduan Mountain Range (Figure 1). The mean annual temperature is approximated as 11.3°C, with the average annual precipitation of 1804 mm. Soils are yellowish‐brown, acidic, with a pH range of 4.2–4.9 (Wu et al. 2012). The forest is representative of moist evergreen broad‐leaved forests, and predominantly composed of species from family of Fagaceae and Lauraceae, including *Castanopsis wattii* (King ex Hook. f.) *A. Camus* , *Lithocarpus hancei* (Benth.) Rehder, *Machilus gamblei* King ex Hook. f. and *Cinnamomum chago* B. S. Sun & H. L. Zhao. In the mature to over‐mature stage, the forest community height ranges from 20 to 25 m, and its canopy is dense, with a vegetation coverage of over 0.85 (Yunnan Vegetation Editorial Committee 1987).

### 2.2 Forest Inventory, Functional Traits and Environmental Factors

Thirty 20 m × 20 m plots were randomly set along an altitudinal gradient from 2450 to 2650 m within the reserve, with the average spacing between adjacent plots exceeding 200 m. We identified species and measured DBH and height for all healthy trees (DBH ≥ 1 cm) in the plots. Tree species were identified following the taxonomic nomenclature of *Flora of China* (https://www.iplant.cn/) and *Flora of Yunnan* (Wu 1977–2006). A total station instrument was used to measure the relative heights of the corners and the center of 30 plots to facilitate subsequent calculations of topographic features. Each plot was divided into four quadrants, the surface litter layer from the center of the quadrants was removed, then soil samples were gathered at a depth ranging from 0 to 20 cm and homogenized thoroughly. Edaphic factors were determined with reference to *Agricultural Chemical Analysis in Soil* (Carter and Gregorich 2007). Five functional traits related to forest ecosystem carbon accumulation were determined as follows: specific leaf area was calculated as the ratio of fresh‐leaf area (single side) to leaf dry mass and leaf nitrogen, phosphorus, potassium and carbon concentration were determined via the kjeldahl method, molybdenum blue colorimetric method, flame photometry method and potassium dichromate method, respectively (Cornelissen et al. 2003).

### 2.3 CWMs, Functional and Phylogenetic Diversity Metrics

Functional diversity metrics were calculated based on above measured functional traits. Community‐weighted mean (CWM) values for each individual trait were calculated separately for the overstory and understory layers.

(1) $$\mathrm{CWM}=\sum_{i=1}^{n}\mathrm{wi}\times \text{traiti}$$

where *wi* is the relative abundance of species *i*; traiti is the trait value of species *i*; and *n* is the total number of species in the community.

Three key functional diversity indices were calculated as follows (Vargas‐Larreta et al. 2021):

(2) $$\begin{array}{c} \text{FDis}=\sum_{i=1}^{s}\mathrm{wi}\times \mathrm{zi} \end{array}$$

where *S* is the total number of species in the community; *wi* is the abundance weight of species *I*; and *zi* is the distance between the functional trait vector of species *i* and the community‐weighted centroid *c*.

(3) $$\begin{array}{c} \text{FRic}=\frac{\left({\mathrm{SF}}_{\mathrm{ic}}\right)}{R_{c}} \end{array}$$

where FRic is the functional richness of trait *c* in community *i*; SFic is the ecological niche space occupied by species in the community; and *Rc* is the absolute eigenvalue of different traits across communities

(4) $$\begin{array}{c} \text{FDiv}=\frac{2}{\pi }\arctan\left\{5\times \sum_{i=1}^{N}\left[{\left(\ln C_{i}-\ln\bar{x}\right)}^{2}\times A_{i}\right]\right\} \end{array}$$

where *C_i_* is the value of the i‐th functional trait; *A_i_* is the relative abundance of the *i*‐th functional trait; $\ln\bar{x}$ is the weighted mean of the natural logarithm of species trait values; *N* is the total number of functional traits considered. Above analysis were performed in R packages “FD” (Laliberté and Legendre 2010).

**FIGURE 2:**
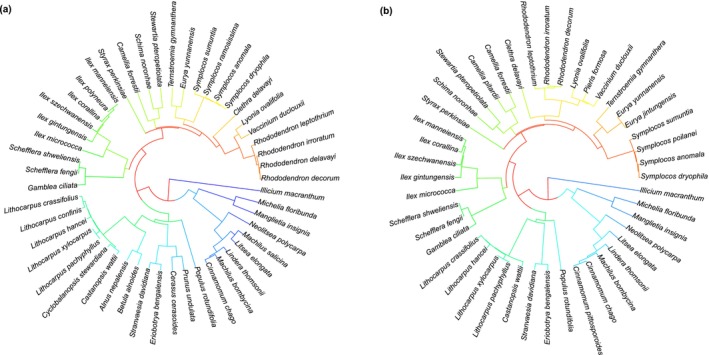
Phylogenetic trees among tree species across forest strata in the study area were reconstructed using the Maximum Likelihood (ML) method, based on a concatenated alignment of four molecular markers: *RbcL*, *matK*, *trnH–psbA*, and ITS. Panel (a) presents the ML phylogeny of 48 overstory tree species; panel (b) shows the ML phylogeny of 44 understory tree species. Phylogenetic diversity (PD) indices for each forest stratum were subsequently calculated from these phylogenies.

Phylogenetic trees were constructed for 48 overstory tree species and 44 understory tree species (Figure 2). Genomic DNA was extracted from fresh leaf tissue using the Plant Genomic DNA Extraction Kit (Tiangen Biotech Co. Ltd., Beijing, China) following the manufacturer's protocol. Four DNA regions were targeted: the chloroplast fragments *rbcL*, *matK*, and *trnH–psbA*, and the nuclear ribosomal internal transcribed spacer (ITS). Multiple sequence alignments were performed using MEGA software (Yang et al. 2014), and the aligned sequences of the four markers were concatenated to construct a supermatrix. A maximum likelihood (ML) phylogeny was inferred, and node support was evaluated using non‐parametric bootstrap analysis. To address discrepancies in branch length units across raw phylogenies, branch lengths were standardized using specific parameters (e.g., genetic distance ‘dN’ or evolutionary rate ‘rate’) implemented in the R package ‘V.PhyloMaker2’. Based on the constructed phylogeny, the following indices were calculated using the ‘picante’ package in R:

(5) $$\begin{array}{c} \mathrm{MPD}=\frac{2}{n\left(n-1\right)}\sum_{i=1}^{n-1}\sum_{j=i+1}^{n}d\left(s_{i},s_{j}\right) \end{array}$$

(6) $$\begin{array}{c} \text{MNTD}=\frac{1}{n}\sum_{i=1}^{n}\min_{j\neq i}d\left(s_{i},s_{j}\right) \end{array}$$

where *n* is the total number of species in the community; *d*(*si*, *sj*) is the path length between species *si* and *sj* on the phylogenetic tree; and min_*j≠i*_ *d*(*si*, *sj*) represents the shortest distance from species *si* to any other species in the community.

(7) $$\begin{array}{c} \mathrm{PSR}=n\times \mathrm{PSV} \end{array}$$

where *n* is the total number of species in the community; phylogenetic species variability (PSV) summarizes the degree of phylogenetic relatedness among species within a community, ranging from 0 to 1. Values approaching 1 indicate that the species are less phylogenetically related (i.e., more distantly related). The specific calculation method follows Helmus et al. (2007). All diversity measures were calculated using the “picante” packages (Kembel and Ackerly 2011) in R.

### 2.4 Aboveground Biomass Calculation

A set of allometric equations was used to estimate AGB, including five species‐specific equations for dominant species and a universal equation for other tree species. The species‐specific allometric equations for the five dominant species were established during the establishment of the nature reserve in the 1980s, and the detailed estimation method followed Qiu et al. (1984). For example, the AGB of *Castanopsis wattii* (King ex Hook. f.) *A. Camus* was calculated using the equation: $\mathrm{AGB}=0.03086\times {\left(D^{2}\times H\right)}^{0.9696}$, (*r* ^2^ = 0.998), where *D* and *H* represent DBH and tree height, respectively. A total of 35 sample trees representing 14 common tree species in the study area (with DBH ranging from 1 to 100 cm) were selected to develop a general allometric biomass equation applicable to all tree species in the study area. The equation is as follows: $W=a{\left(D^{2}H\right)}^{b}$, where *W* is the biomass of a species; *a* and *b* are constants; *D* and *H* represent DBH and tree height, respectively (Liu et al. 2002). The overstory AGB comprises the total biomass of individual trees with a DBH ≥ 9 cm in the forest community, and the understory AGB refers to the total biomass of trees with a DBH < 9 cm (Zhang et al. 2016).

### 2.5 Statistical Analyses

Principal component analysis (PCA) was performed on the selected topographic and edaphic variables in order to reduce the dataset dimensionality. The resulting principal components—Soil_PC1, Soil_PC2, Topo_PC1, and Topo_PC2—were used to represent local soil and topographic conditions in subsequent analyses. The relationship between biodiversity indices and AGB was evaluated by applying partial least squares (PLS) regression. First, all explanatory variables were assembled into a predictor matrix, which was then regressed against AGB. Next, the “shaving” function was utilized to remove the less informative predictors of AGB. Second, a refined model was established based on the variable selection outcomes, and the variable importance for projection (VIP) of the retained predictors was computed via the “plsreg2” function (Trogisch et al. 2016). Variables were classified according to their VIP values: those with VIP > 1 were considered major contributors, values between 1 and 0.8 indicated medium contributors, and those with VIP < 0.8 were regarded as having negligible contributions (Frameschi et al. 2013). All computations were performed with plsVarSel and plsdepot packages in R (R Core Team 2025). Linear regression models were fitted to depict the relationships between AGB and the informative predictors for both the overstory and understory layers, using the R package “ggplot2”.

## 3 Results

**FIGURE 3:**
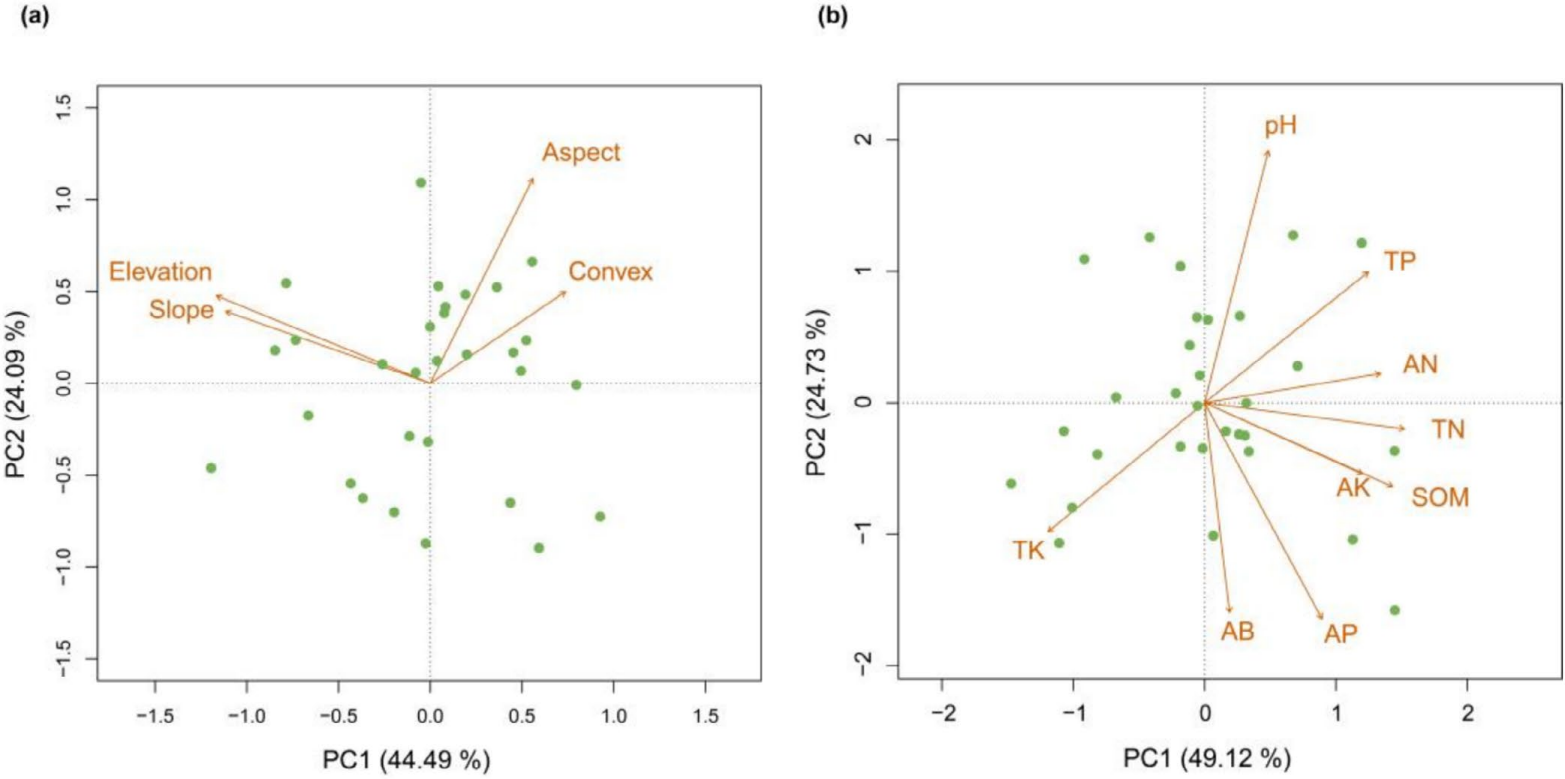
Principal component analysis of topographic and soil factors. (a) PCA of topographic covariates. PC1 and PC2 explain 44.49% and 24.09% of the total topographic variation, respectively. (b) PCA of soil parameters. PC1 and PC2 explain 49.12% and 24.73% of total variance in the selected soil parameters, respectively. AB, available boron (g/kg)AK, available potassium (g/kg); AN, available nitrogen (g/kg); AP, available phosphorus (g/kg); SOM, soil organic matter (g/kg); TK, total potassium (g/kg); TN, total nitrogen (g/kg); TP, total phosphorus (g/kg).

The first two PCA axes accounted for 44.49% and 24.09% of the topographic variance; Topo_PC1 was determined by elevation and slope, while Topo_PC2 was determined by aspect (Figure 3). Soil_PC1 explained 49.12% of total variance in the selected soil parameters, and it was primarily related to available potassium, available nitrogen, soil organic matter, total nitrogen, total phosphorus, and total potassium. Soil_PC2 accounted for 24.73% of the total variability and was determined by available boron, available phosphorus, and pH (Figure 3).

**FIGURE 4:**
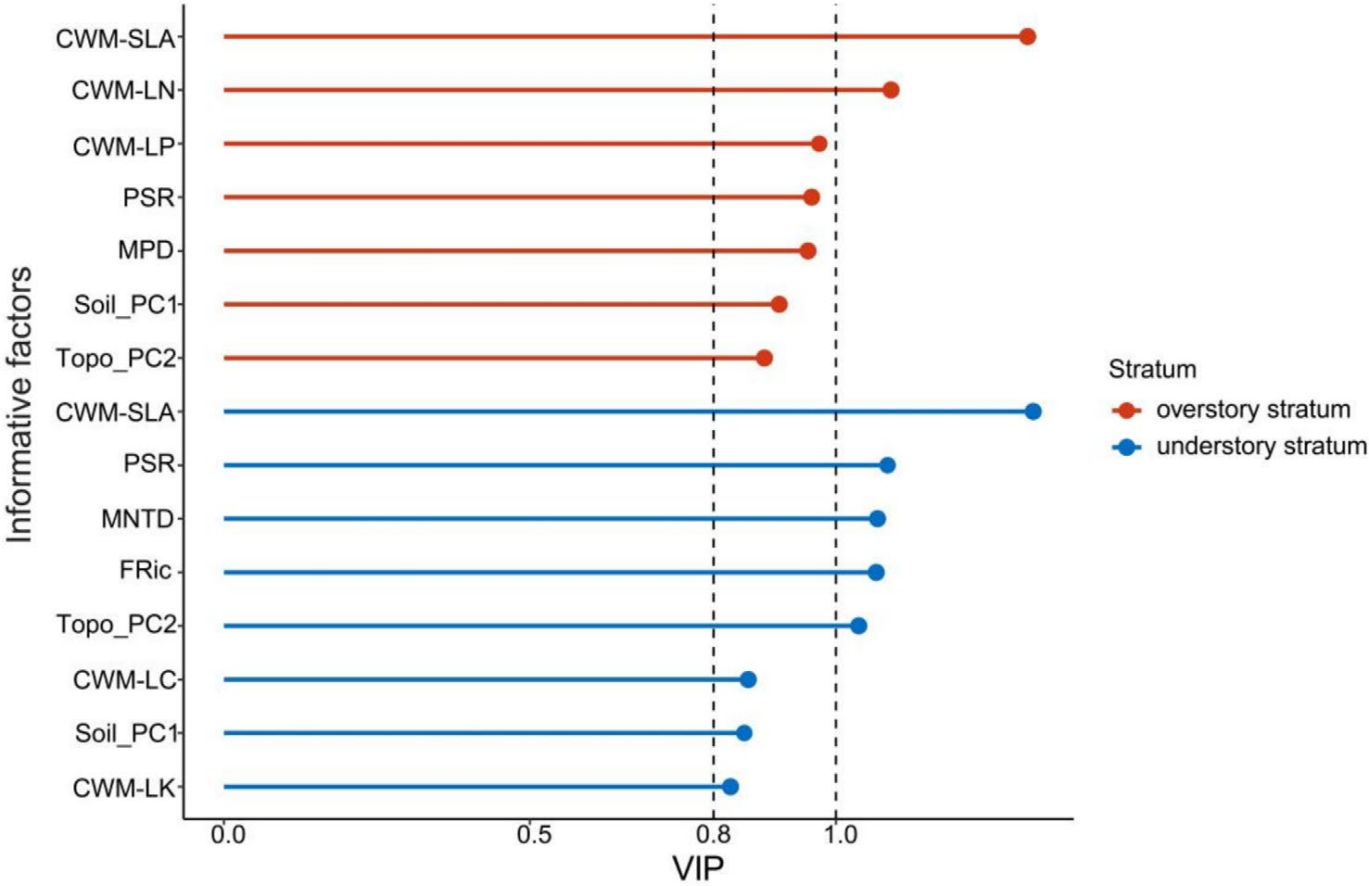
Variable importance in projection (VIP) scores derived from partial least squares (PLS) regression for predictors of AGB. Variables with VIP > 1.0 are important contributors to AGB, while those with 0.8 < VIP < 1.0 are regarded as moderate predictors. Red bars represent the overstory stratum, and blue bars represent the understory stratum.

The AGB of overstory and understory tree layers was 343.47 ± 138.29 Mg/ha and 15.16 ± 10.27 Mg/ha, respectively. CWM‐SLA, CWM‐LN, CWM‐LP, MPD, PSR, Soil_PC1 and Topo_PC2 were identified as informative predictors for the overstory AGB, among which CWM‐SLA and CWM‐LN were of most importance (VIP > 1.0); MPD, PSR, CWM‐LP, Soil_PC1 and Topo_PC2 were less important (0.8 < VIP < 1.0) (Figure 4). CWM‐SLA, MNTD, PSR, FRic and Topo_PC2 were the most important predictors for the understory AGB (VIP > 1.0); while CWM‐LC, CWM‐LK and Soil_PC2 were moderate predictors for it (0.8 < VIP < 1.0) (Figure 4).

**FIGURE 5:**
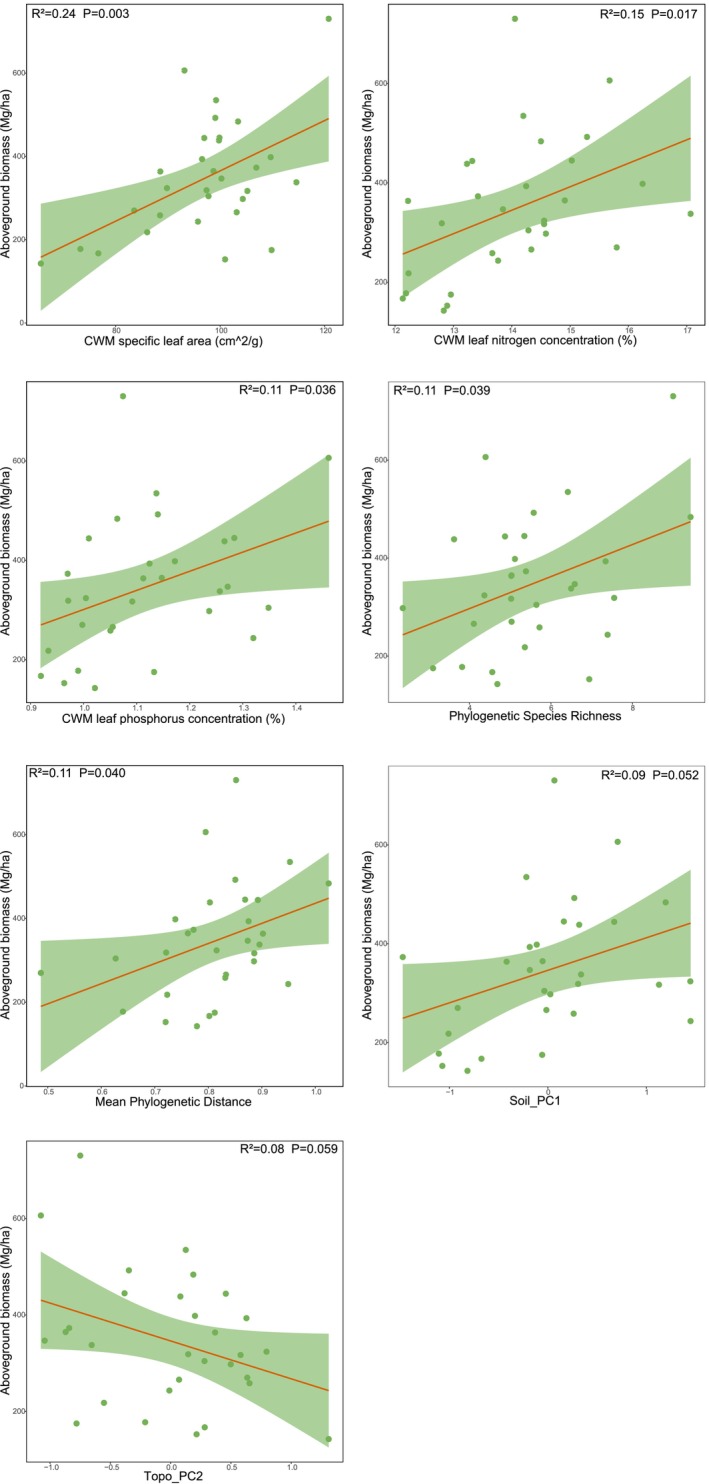
Linear regression relationships between overstorey AGB and informative predictors. The orange solid line represents the linear regression fit, the light green shaded area represents the 95% confidence interval, and the green points denote the observed values for each sample.

**FIGURE 6:**
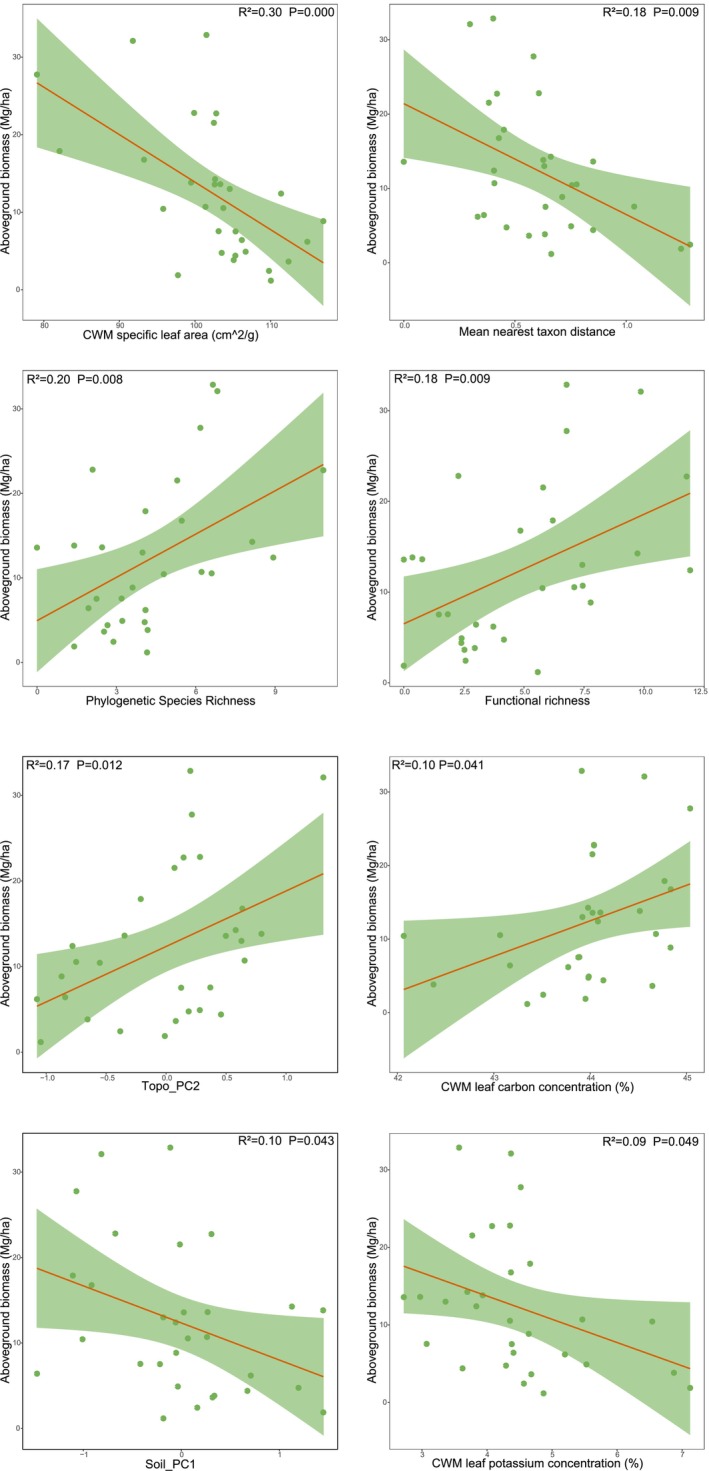
Linear regression relationships between understory AGB and informative predictors. The orange solid line represents the linear regression fit, the light green shaded area represents the 95% confidence interval, and the green points denote the observed values for each sample.

Overstory AGB significantly increased with higher CWM‐SLA (*R* ^2^ = 0.24, *p* = 0.003), CWM‐LN (*R* ^*2*^ = 0.15, *p* = 0.017), CWM‐LP (*R* ^*2*^ = 0.11, *p* = 0.036), PSR (*R* ^2^ = 0.11, *p* = 0.039), and MPD (*R* ^2^ = 0.11, *p* = 0.040). Soil_PC1 (*R* ^2^ = 0.09, *p* > 0.05) and Topo_PC2 (*R* ^2^ = 0.08, *p* > 0.05) showed marginally positive and negative correlation with overstory AGB, respectively (Figure 5). In contrast, understory stratum exhibited higher AGB when characterized by greater PSR (*R* ^2^ = 0.20, *p* = 0.008) and FRic (*R* ^2^ = 0.18, *p* = 0.009). In addition, understory AGB was positively associated with CWM‐LC (*R* ^2^ = 0.10, *p* = 0.041) and Topo_PC2 (*R* ^2^ = 0.17, *p* = 0.012), and decreased with increasing CWM‐SLA (*R* ^*2*^ = 0.30, *p* < 0.001) and CWM‐LK (*R* ^2^ = 0.09, *p* = 0.049), MNTD (*R* ^2^ = 0.18, *p* = 0.009) and Soil_PC1 (*R* ^2^ = 0.10, *p* = 0.043) (Figure 6).

## 4 Discussion

### 4.1 Influences of CWM Traits on AGB

In our study, high overstory AGB was mainly driven by the tree species with high CWM‐SLA, CWM‐LN, and CWM‐LP, reflecting their ability to maximize resource use efficiency through rapid acquisition strategies. Species with shorter leaf lifespans typically maintain high specific leaf area and leaf nitrogen content to efficiently capture light and nutrients (Llerena‐Zambrano et al. 2021). Previous studies have confirmed that traits associated with rapid resource acquisition strategies enhance the competitive ability of tree species and allocate more resources to the aboveground parts (Li et al. 2020; Sun et al. 2023). In addition, elevated leaf phosphorus concentrations promote photosynthetic efficiency and plant growth, providing energy and material support for rapid growth of aboveground plant parts (Feng et al. 2023). Our study revealed that the AGB of the overstory is primarily governed by the functional identity of tree species, providing empirical support for the mass ratio hypothesis or the selection effects.

Contrary to the overstory, understory AGB exhibited a negative correlation with CWM of SLA and LK. Long‐term lack of light severely restricts the growth of understory tree species and shapes their survival strategies (Brenes‐Arguedas et al. 2011). More specifically, understory tree species tend to possess conservative strategies by reducing resource consumption and enhancing stress resistance to adapt to high‐stress environments and maintain their survival and growth (Bartels and Chen 2010; Zhang et al. 2016). Low specific leaf area indicates thicker leaves with higher leaf carbon content, with which tree species can reduce transpiration water loss and photosynthetic structure damage, but enhance resistance to understory shading and pathogen infection (Wright et al. 2004). This is consistent with findings that lower specific leaf area is associated with higher plant survival rates, leading to greater AGB with low resource availability and high biodiversity (Liu et al. 2023). Although high leaf potassium can improve stress resistance in resource‐limited environments, excessive allocation to leaf potassium occupies investment in growth‐related resources (Shi et al. 2024), ultimately impairing AGB accumulation. In combination, our first hypothesis is partly supported: functional identity effectively explains AGB variation across forest layers, but the way functional traits work behind the mass ratio hypothesis may be different between them.

### 4.2 Influence of Functional and Phylogenetic Diversity on AGB Across Forest Strata

Functional diversity and phylogenetic diversity are crucial for explaining variations in forest AGB. At present, there is a paucity of research comparing the relative importance of functional and phylogenetic diversity in ecosystem functions. The few research results indicate that functional diversity has a greater impact on ecosystem functions than phylogenetic diversity (Flynn et al. 2011; Steudel et al. 2016). However, our results are exactly the opposite: phylogenetic diversity is more explanatory of AGB accumulation than functional diversity, whether in the overstory or understory tree layers.

We found that two phylogenetic diversity indices, phylogenetic species richness (PSR) and mean phylogenetic distance (MPD), were important predictors for AGB. Specifically, overstory AGB was higher in groups composed of phylogenetically distant species with high MPD. This pattern could be attributed to the late‐successional stage of the studied forest, where large trees dominate the overstory. For trees at different diameter scales, large trees tended to be phylogenetically divergent (Swenson et al. 2007). As the number of large trees increases, interspecific competition intensifies, which leads to greater geographical separation and phylogenetic divergence (Hou et al. 2017). However, these dominant species still contribute significantly to biomass, thereby establishing a positive MPD‐AGB correlation. As a metric that integrates species richness and evolutionary history (Helmus et al. 2007), phylogenetic species richness (PSR) showed a positive correlation with overstory AGB in our study, suggesting that coexisting species are distantly related and exhibit significant phylogenetic differentiation. Distantly related species have less niche overlap, leading to stronger complementarity in resource utilization (Cadotte 2013), which enhances biomass production.

The functional diversity indices were not included as informative predictors in the model for predicting the overstory AGB, which implies a stronger influence of evolutionary history on AGB than functional diversity. In this context, phylogenetic diversity (PD) can serve as a reliable proxy for functional diversity (FD) (Ali and Yan 2018). Phylogenetic diversity provides more effective predictions for the complex interactions between species in multidimensional ecological niches (Huang et al. 2020). This may be because phylogenetic diversity metrics reflect the diversity of phylogenetically conserved traits related to resource utilization, acquisition, and storage. Traits such as mycorrhizal symbiosis (a mutualistic interaction) and susceptibility to specific pathogens (an antagonistic interaction) exhibit strong phylogenetic conservatism (Souza et al. 2016; Mazzochini et al. 2019). Critically, pathogen transmission pathways reflect the degree of phylogenetic relatedness (Parker et al. 2015). The probability that pathogens are transmitted between phylogenetically close species is greater than that between phylogenetically distant species, with these fungal pathogens not only decreasing plant biomass but also triggering higher species mortality under severe infection (Qin et al. 2020; Yan et al. 2023). Consequently, communities with high phylogenetic diversity maintain higher and more stable biomass productivity.

Phylogenetic species richness (PSR) in the understory was a better predictor of AGB than in the overstory. Understory species exhibit greater evolutionary divergence than overstory species and enhance resource use efficiency through a stronger complementarity effect (Cadotte 2013), thereby maximizing the aboveground biomass productivity. Although functional richness also drives AGB accumulation via the complementarity effect (Roscher et al. 2013), the explanatory power of phylogenetic diversity (PSR; VIP = 1.084) was higher than that of functional diversity (FRic; VIP = 1.065), supporting our second hypothesis. Recent evidence suggests that phylogenetic diversity buffers the impact of environmental fluctuations on ecosystem functioning via the regulation of functional trait composition (Zhang et al. 2026), highlighting its central role in maintaining ecosystem stability. Furthermore, AGB was negatively correlated with mean nearest taxon distance (MNTD), implying that the coexistence of closely related shade‐tolerant species in the understory is more conducive to promoting AGB. Generally, shade tolerance of species is often associated with a conservative resource use strategy (Kembel 2009), which weakens the intensity of interspecific resource competition (van Steijn et al. 2025) and thus facilitates AGB productivity.

## 5 Conclusions

Community‐weighted mean traits (functional identity) were the primary drivers of AGB across forest strata, yet the underlying strategies differed fundamentally between layers: resource‐acquisitive strategies dominated the overstory, whereas conservative strategies prevailed in the understory. These results indicate that it is essential to analyze forest strata separately for a better comprehension of the relationship between biodiversity and AGB accumulation in subtropical forests. The niche complementary effects, which were characterized by both functional and phylogenetic diversity, also largely determined AGB accumulation across tree layers. When functional diversity was not incorporated as a valid predictor of AGB in the overstory, phylogenetic diversity replaced it to effectively explain variation in AGB through complementary effects. These findings highlight the need for further exploration of the relative importance of functional diversity and phylogenetic diversity in specific ecosystem processes in future studies, providing a scientific basis for the protection and scientific management of subtropical forest ecosystems.

## Author Contributions

**Qingping Li:** conceptualization (lead), data curation (equal), formal analysis (equal), investigation (equal), visualization (equal), writing – original draft (lead). **Yizhi Wang:** conceptualization (supporting), supervision (supporting). **Xiuqin Ci:** project administration (equal), resources (equal), supervision (equal). **Zhiyun Lu:** project administration (lead), resources (lead), supervision (equal). **Yuanjie Xu:** conceptualization (equal), data curation (equal), formal analysis (equal), funding acquisition (lead), supervision (lead), visualization (equal), writing – review and editing (lead).

## Funding

This work was supported by Ten Thousand Talents Program of Yunnan Province, YNWR‐QNBJ‐2019‐244, XDYC‐QNRC‐2024‐252. National Natural Science Foundation of China, NSFC 32460295.

## Conflicts of Interest

The authors declare no conflicts of interest.

## Data Availability

The dataset used for analyses accompanies this paper as a Supporting Information. More information about the dataset is available upon request to the authors.
